# A [Fourth] Analysis of Cases of Tetanus Treated in Home Hospitals (during October, November, and December, 1916)

**Published:** 1918-01

**Authors:** 


***A [Fourth] Analysis of Cases of Tetanus Treated in Home Hospitals (during October, November, and December, 1916).*** By Sir David BRUCE. Abs. from the *Lancet*, Sept. 15, 1917.

In the group of 100 cases here analysed the mortality has fallen to 31 o/o. There were 61 cases of generalized tetanus with a mortality of 34.4 o/o, and 28 cases of localized tetanus, all of which recovered. The author can discover no evidence for or against the intrathecal route or any conclusive evidence as to the curative value of antitoxin. The general diminution in the incidence of tetanus, and the increase in the average of recovery is ample testimony to the value of preventive inoculation.

